# Daylight saving time as a potential public health intervention: an observational study of evening daylight and objectively-measured physical activity among 23,000 children from 9 countries

**DOI:** 10.1186/1479-5868-11-84

**Published:** 2014-10-23

**Authors:** Anna Goodman, Angie S Page, Ashley R Cooper, for the International Children’s Accelerometry Database (ICAD) Collaborators

**Affiliations:** Faculty of Epidemiology and Population Health, London School of Hygiene and Tropical Medicine, Keppel Street, London, WC1E 7HT UK; Centre for Exercise, Nutrition and Health Sciences, University of Bristol, Bristol, UK; National Institute for Health Research, Bristol Biomedical Research Unit in Nutrition, Diet and Lifestyle, Bristol, UK

**Keywords:** Child, Adolescent, Physical activity, Day length, Seasons

## Abstract

**Background:**

It has been proposed that introducing daylight saving measures could increase children’s physical activity, but there exists little research on this issue. This study therefore examined associations between time of sunset and activity levels, including using the bi-annual ‘changing of the clocks’ as a natural experiment.

**Methods:**

23,188 children aged 5–16 years from 15 studies in nine countries were brought together in the International Children’s Accelerometry Database. 439 of these children were of particular interest for our analyses as they contributed data both immediately before and after the clocks changed. All children provided objectively-measured physical activity data from Actigraph accelerometers, and we used their average physical activity level (accelerometer counts per minute) as our primary outcome. Date of accelerometer data collection was matched to time of sunset, and to weather characteristics including daily precipitation, humidity, wind speed and temperature.

**Results:**

Adjusting for child and weather covariates, we found that longer evening daylight was independently associated with a small increase in daily physical activity. Consistent with a causal interpretation, the magnitude of these associations was largest in the late afternoon and early evening and these associations were also evident when comparing the same child just before and just after the clocks changed. These associations were, however, only consistently observed in the five mainland European, four English and two Australian samples (adjusted, pooled effect sizes 0.03-0.07 standard deviations per hour of additional evening daylight). In some settings there was some evidence of larger associations between daylength and physical activity in boys. There was no evidence of interactions with weight status or maternal education, and inconsistent findings for interactions with age.

**Conclusions:**

In Europe and Australia, evening daylight seems to play a causal role in increasing children’s activity in a relatively equitable manner. Although the average increase in activity is small in absolute terms, these increases apply across all children in a population. Moreover, these small effect sizes actually compare relatively favourably with the typical effect of intensive, individual-level interventions. We therefore conclude that, by shifting the physical activity mean of the entire population, the introduction of additional daylight saving measures could yield worthwhile public health benefits.

**Electronic supplementary material:**

The online version of this article (doi:10.1186/1479-5868-11-84) contains supplementary material, which is available to authorized users.

## Background

Physical activity confers substantial physical and mental health benefits in children [1–5], but most children around the world do not meet current activity guidelines [6]. For children as for adults, successfully promoting physical activity is likely to require both individual-level and population-level interventions [7]. The latter are important because, following the insights of Geoffrey Rose [8], even a small shift in a population mean can yield important public health benefits.

One potential population-level measure which has received some policy attention in recent years concerns the introduction of additional daylight saving measures [9]. Although the total number of hours of daylight in the day is fixed, many countries modify when those hours fall by ‘changing the clocks’ – for example, putting the clocks forward in the summer to shift daylight hours from the very early morning to the evening. Recent decades have seen recurrent political debates surrounding daylight saving measures in several countries. For example, several Australian states have held repeated referenda on the topic, and the issue even spawned the creation in 2008 of the single-issue political party ‘Daylight Saving for South East Queensland’. Similarly a Bill was debated in the British Parliament between 2010 and 2012 which proposed to shift the clocks forward by an additional hour year round. This change would have given British children an estimated average of 200 extra waking daylight hours per year [10], and the logo of the associated civil society campaign depicted children playing outdoors in the evening sunlight. The Bill’s accompanying research paper listed “increased opportunities for outdoor activity” alongside other potential health and environmental benefits, such as reducing road traffic crashes and cutting domestic energy use [11]. A similar argument about leisure-time activity has featured in the Australian debate [12].

The British Bill’s research paper did not, however, cite any evidence to support its claims about physical activity, and nor does much evidence exist regarding likely impacts on children. Many studies have certainly reported that children’s physical activity is generally higher in the summer than in the winter, as reviewed in [13–15]. Very few studies, however, examine whether seasonal differences persist after adjustment for weather conditions, or whether the seasonal patterning of physical activity across the day is consistent with a causal effect of evening daylight. One study which did examine these issues in detail found that seasonal differences in physical activity were greatest in the late afternoon and early evening, which is what one would expect if time of sunset did play a causal role [16]. This study had some major limitations, however, including its small sample size (N = 325), its restriction to a single setting in south-east England, and its failure to adjust for temperature.

This paper therefore revisited this question in a much larger, international sample. Our first broad aim was to test the hypotheses that (i) longer evening daylight is associated with higher total physical activity, even after adjusting for weather conditions; and (ii) these overall differences in physical activity are greatest in the late afternoon and early evening. Given our uniquely large sample size, we were also able to use countries’ bi-annual changing of the clocks as a natural experiment, i.e. as an event or intervention not designed for research purposes but which can nevertheless provide valuable research opportunities [17]. Specifically, we tested the hypothesis that the same child measured immediately before and immediately after the clocks changed would be more active on the days where sunset had been moved an hour later. Our second broad aim was to examine whether any associations between evening daylight and activity levels differed by study setting, sex, age, weight status or socio-economic position.

## Methods

### Study design

The International Children’s Accelerometry Database (http://www.mrc-epid.cam.ac.uk/research/studies/icad/) was established to pool objectively-measured physical activity data from studies using the Actigraph accelerometer in children worldwide. The aims, design and methods of ICAD have been described in detail elsewhere [18]. Formal data sharing agreements were established and all partners consulted their individual research board to confirm that sufficient ethical approval had been attained for contributing data.

### Participants

The full ICAD database pools accelerometer data from 20 studies conducted in ten countries between 1997 and 2009 [18]. In this paper, we excluded four studies which focussed on pre-school children and one study for which date of measurement was not available. We used baseline data from all of the 15 remaining studies, plus follow-up measurements in the seven longitudinal studies and one natural experimental study (Additional file 1: Table A1). We also used follow-up measurements from the control group of one of the two randomised controlled trials, as for this study it was possible to distinguish intervention and control groups.

Among 23,354 individuals aged 5–16 years old in the 15 eligible studies, we excluded 1.7% of measurement days (0.7% of individuals) because of missing data on age, sex, weight status or weather conditions. Our resulting study population consisted of 23,188 participants who between them provided 158,784 days of valid data across 31,378 time points (Table 1). Although our full study population included children providing data from any part of the year, one of our analyses was limited to 439 children who were sampled during a week which spanned the clock change (51% female, age range 5–16, 1830 measurement days).

**Table 1:** **Descriptive characteristics of study participants**

		N (%) participants	N (%) valid days
Full sample		23,188 (100%)	158,784 (100%)
Sex	Male	8819 (38%)	62,745 (40%)
	Female	14,369 (62%)^†^	96,039 (60%)
Age	5-6 years	1800 (8%)	7855 (5%)
	7-8 years	711 (3%)	4963 (3%)
	9-10 years	5769 (25%)	30,702 (19%)
	11-12 years	9616 (41%)	61,352 (39%)
	13-14 years	4206 (18%)	46,530 (29%)
	15-16 years	1086 (5%)	7382 (5%)
Country [No. studies]	Australia [N = 2]	2459 (11%)	18,679 (12%)
	Brazil [N = 1]	453 (2%)	1577 (1%)
	Denmark [N = 2]	2031 (9%)	11,030 (7%)
	England [N = 4]	10,284 (44%)	83,420 (53%)
	Estonia [N = 1]	656 (3%)	2537 (2%)
	Madeira [N = 1]	1214 (5%)	4899 (3%)
	Norway [N = 1]	384 (2%)	1459 (1%)
	Switzerland [N = 1]	404 (2%)	2569 (2%)
	United States [N = 2]	5303 (23%)	32,614 (21%)
Weight status	Normal/underweight	17,573 (76%)	121,350 (76%)
	Overweight	4116 (18%)	27,967 (18%)
	Obese	1499 (6%)	9467 (6%)
Mother’s education	Up to high school	7422 (48%)	54,547 (48%)
	College/vocational	2656 (17%)	19,352 (17%)
	University level	5251 (34%)	38,723 (34%)

For individuals measured more than once, the first column gives age and weight status at baseline while the second column gives age and weight status during the measurement period in question. Numbers add up to less than the total for mother’s education because this variable was only collected in 11 of the 15 studies, and was also subject to some missing data within those 11 studies (see Additional file 1: Tables A1 and A2). ^†^Proportion of girls 52% after excluding one large American study that measured girls only.

### Measurement of physical activity

All physical activity measurements were made with uniaxial, waist-worn Actigraph accelerometers (models 7164, 71256 and GT1M); these are a family of accelerometers that have been shown to provide reliable and valid measurement of physical activity in children and adolescents [19–21]. All raw accelerometer data files were re-analysed to provide physical activity outcome variables that could be directly compared across studies (see [18] for details). Data files were reintegrated to a 60 second epoch where necessary and processed using commercially available software (KineSoft v3.3.20, Saskatchewan, Canada). Non-wear time was defined as 60 minutes of consecutive zeros allowing for 2 minutes of non-zero interruptions [22].

We restricted our analysis of activity data to the time period 07:00 and 22:59, and defined a valid measurement day as one recording at least 500 minutes of wear time during this time period (18% days excluded as invalid). When examining the pattern of physical activity across the day, we only included hours with at least 30 minutes of measured wear time. Each participating child provided an average of 5.1 days across the week in which they were measured (range 1–7); we did not require a minimum number of valid days of accelerometer data per child because days, not children, were our primary units of analysis.

Although we sought to limit our analyses to activity during waking hours, we unfortunately lacked reliable data on the time children went to sleep or woke up. While most children took their accelerometers off to sleep, on 6% of days there was evidence of overnight wear, defined as ≥5 minutes of weartime between 1:00 and 04:59. On these days, we assumed the child was in fact sleeping during any hour between 21:00 and 07:59 for which the mean accelerometer counts per minute (cpm) was below 50. Mean cpm values of under 50 were observed for 90% of hours recorded between 03:00 and 03:59 but only 3% of hours recorded between 19:00 and 19:59, suggesting this cut-point provided a reasonable proxy for sleeping time among children for whom we had reason to suspect overnight wear. Our findings were unchanged in sensitivity analyses which instead used thresholds of 30 cpm or 100 cpm to exclude suspected sleeping time, or which excluded altogether the 6% of days with suspected overnight wear.

Our pre-specified primary outcome measure was the child’s average counts per minute. Substantive findings were similar in sensitivity analyses which instead used percent time spent in moderate-to-vigorous physical activity (MVPA), defined either as ≥3000 cpm [23] or ≥2296 cpm [24]. For our key findings, we present these MVPA results (using the ≥3000 cpm cut-off) alongside the results for mean cpm. In order to facilitate interpretation of these MVPA results, we additionally convert the observed percentage times into approximate absolute minutes on the assumption of a 14-hour average waking day [25].

### Time of sunset and covariates

For each day of accelerometer wear, we used http://www.timeanddate.com to assign time of sunset on that specific date in the city in which, or nearest which, data collection took place. We also used the date and the city of data collection to assign six weather variables to each day: total precipitation across the day, mean humidity across the day, maximum daily wind speed, mean daily temperature, maximum departure of temperature above the daily mean, and maximum departure of temperature below the daily mean. We accessed these data using Mathematica 9 (Wolfram Research), which compiles daily information from a wide range of weather stations run by states, international bodies or public-private partnerships [26]. The correlation between hour of sunset and mean temperature was moderately but not prohibitively high (r = 0.59), while correlations with other weather covariates were modest (r < 0.30).

The child’s height and weight were measured in the original studies using standardized clinical procedures, and we used these to calculate body mass index (kg/m^2^). Participants were categorized as underweight/normal weight, overweight or obese according to age and sex-specific cut points [27]. Maternal education was assessed in 11/15 studies, and was re-coded to distinguish between ‘high school or lower’ education versus ‘college or university’ education (Additional file 1: Table A2).

### Statistical analyses

Both time of sunset and weather vary between individual days, and we therefore used days not children as our units of analysis. We adjusted for the clustering of days within children using robust standard errors. All analyses used Stata 13.1.

To address our first aim, we fit linear regression models with the outcome being daily or hourly activity cpm. Time of sunset was the primary explanatory variable of interest, with adjustment for study population, age, sex, weight status, day of the week and the six weather covariates. When using the changing of the clocks as a natural experiment, we restricted our analyses to the 439 children with at least one valid school day measurement both in the week before and in the week after the clocks changed (e.g. Wednesday, Thursday and Friday before the clocks changed and Monday and Tuesday afterwards).

To address our second aim, we calculated the adjusted effect size of evening daylight separately for each study population. We used forest plots to present the fifteen resulting effect sizes, together with an I^2^ value representing between-study heterogeneity and with an overall pooled effect size estimated using random effects meta-analysis [28]. We sometimes converted pooled estimates into standardised effect sizes by dividing by the standard deviation of activity cpm for the population in question. We then proceeded to fit interaction terms between evening daylight and the four pre-specified characteristics of sex, age, weight status and maternal education. These four characteristics were selected *a priori* as characteristics that we felt to be of interest and that were relatively consistently measured across the ICAD studies. We fit these interaction terms after stratifying by study population, and calculated I^2^ values and pooled effect sizes. When examining interactions with age, we restricted our analyses to children aged 9–15 as most measurement days (91%) were of children between these ages.

## Results

The characteristics of the participants are summarised in Table 1. Of the measurement days, 66% were schooldays and 38% of days had no precipitation. The average daily temperature was 12°C (range -21 to 33°C, inter-quartile range 7 to 16°C). Mean daily weartime was 773 minutes (12.9 hours), and this was similar regardless of time of sunset (e.g. regression coefficient +1.40 minutes for days with sunset 18:00–19:59 versus pre-18:00 after adjusting for study population, age and sex; and -2.5 minutes for days with sunset post-20:00 versus pre-18:00).

### Evening daylight and overall activity levels

**Figure 1:**
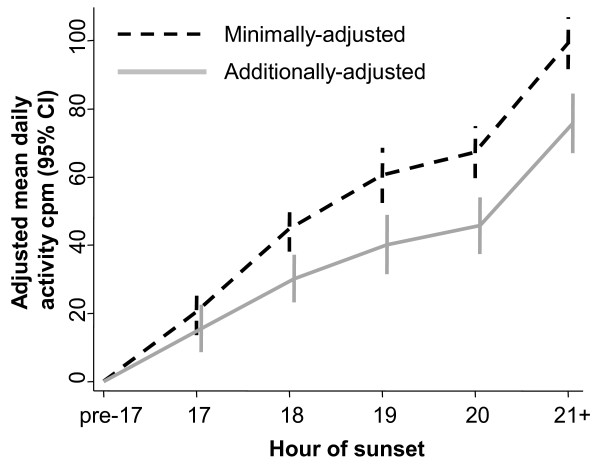
**Association between time of sunset and total daily activity.** CI = confidence interval, cpm = counts per minute. Analysis based on 158,784 measurement days from 23,188 children from 15 studies in 9 countries. Minimally-adjusted analyses adjust for age, sex and study population; additionally-adjusted analyses also include day of the week, weight status and (most importantly) the six weather covariates. Hour of sunset is rounded down, e.g. ‘18’ covers ‘18:00–18:59’, and the reference group is sunset before 17:00.

A later hour of sunset (i.e. extended evening daylight) was associated with increased daily activity across the full range of time of sunset, and this association was only partly attenuated after adjusting for the six weather covariates (Figure 1). Here and for all findings reported subsequently, substantive findings were similar in sensitivity analyses which instead used percent time spent in MVPA. The adjusted difference in overall daily activity between days with sunset after 21:00 vs. before 17:00 was 75 cpm (95% CI 67, 84). The equivalent difference for percent daily time in MVPA was 0.72% (95% CI 0.60, 0.84) using the ≥3000 cpm cut-point, which translates into around 6 minutes. To put the values on the y-axis in context, participants had a mean daily activity count of 560 cpm (649 in boys, 503 in girls), and spent an average of 4.0% of their day, or 33 minutes, in MVPA (5.2%/43 minutes in boys, 3.1%/26 minutes in girls). The adjusted differences between the days with more versus less evening daylight are therefore modest but not trivial in relation to children’s overall activity levels.

### Evening daylight and the patterning of activity across the day

**Figure 2:**
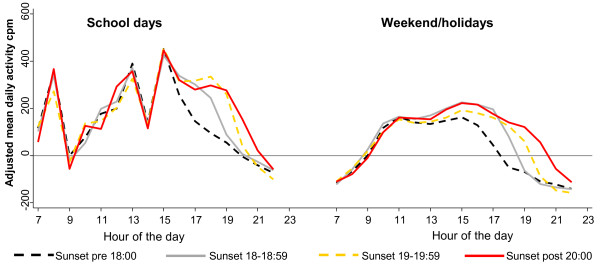
**Adjusted mean activity counts per minute across the hours of the day, according to the time of sunset.** cpm = counts per minute. Analysis based on 158,784 measurement days from 23,188 children from 15 studies in 9 countries. Analyses adjust for study population, age, sex, day of the week, weight status and the six weather variables, with a reference group of 09:00 on days with sunset before 18:00. Hours are rounded down, e.g. ‘18’ covers ‘18:00–18:59’. Confidence intervals not presented as they are generally too narrow to be clearly visible: Additional file 1: Figure A1 includes a version of this graph with confidence intervals.

**Figure 3:**
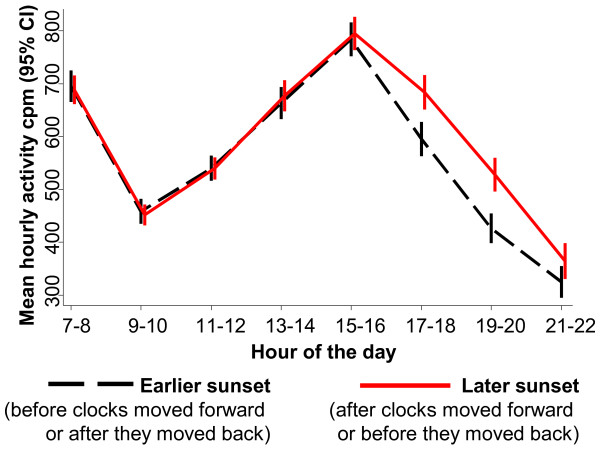
**Mean physical activity across the hours of the day, comparing children either side of the changing of the clocks.** CI = confidence interval, cpm = counts per minute. Analysis based on 1830 schooldays from 439 children from 11 studies in 9 countries. Analyses restricted to children with at least one valid schoolday measurement day both before and after the clocks changed; to increase power, data from across the spring and autumn clock changes are pooled. Hours are grouped into two-hour time periods to increase power and are rounded down, e.g. ‘7-8’ covers ‘07:00–08:59’. Adjustment was not essential as each child serves as his or her own control, but the results were similar in adjusted analyses.

Consistent with a causal interpretation, hour-by-hour analyses indicated that it was in the late afternoon and evening that the duration of evening daylight was most strongly associated with hourly physical activity levels (Figure 2). This was true on both schooldays and weekend/holiday days, with the period of the day when physical activity fell fastest corresponding to the timing of sunset (e.g. falling fastest between 18:00 and 19:00 on days when the sun also set between those hours). Similarly, when comparing the subsample of 439 children who were measured on schooldays immediately before and immediately after the changing of the clocks, there was strong evidence that children were more active during the evening of the days with later sunset (Figure 3). Between 17:00 and 20:59 the mean increase in physical activity on the days with later sunset was 94 cpm per hour (95% CI 62, 125); the equivalent increase in percent of time spent in MVPA was 0.84% (95% CI 0.40%, 1.28%) or 2.0 minutes.

Importantly, Figure 2 and Figure 3 show no association between hour of sunset and activity levels in the morning, and generally no association in the early afternoon (with the exception of a modest effect on weekend/holiday days as early as 14:00 in Figure 2). This suggests that the association between evening daylight and physical activity cannot readily be explained by residual confounding by weather conditions, since any effects of weather would generally be expected to operate more evenly across the day [16]. These findings also provide no suggestion that later sunrise is associated with reduced activity in the morning, including on days when the sun set before 18:00 and on which the average time of sunrise was not until 07:27 (inter-quartile range 07:05 to 07:54).

### Examining differences by place, sex, age, weight and maternal education

**Figure 4:**
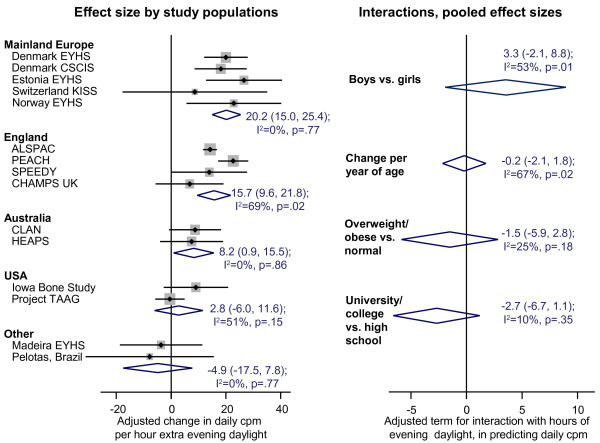
**Association between evening daylight and physical activity across study populations, and pooled effect sizes for interactions by sex, age, weight status and maternal education.** cpm = counts per minute. Analysis based on 23,188 children from 15 studies in 9 countries, except for the comparison of maternal education which is based on 15,563 children in 11 studies in 8 countries (see Additional file 1: Tables A1 and A2 for details of studies providing maternal education data). On the left, random-effects pooled estimates are presented by country/region, together with 95% confidence intervals. Points to the right of the line indicate that longer evening daylight is associated with increased mean daily cpm, points to the left indicate the reverse. On the right, pooled effect sizes and 95% confidence intervals are shown following tests for interaction, with the adjusted interaction term representing the difference that the interaction variable (e.g. sex) makes to the effect size for evening daylight upon total daily activity measured in cpm. For interaction terms stratified by study population see the Additional file 1: Figures A3 and A4.

As shown in Figure 4, there was strong evidence that the association between evening daylight and physical activity varied systematically between settings (I^2^ = 75%, p < 0.001, for overall heterogeneity between the 15 studies). Specifically there was relatively consistent evidence that evening daylight was associated with higher average physical activity in mainland Europe, England and (to a lesser extent) Australia. The pooled point estimates of the increase in daily mean activity in these three settings were 20.2 cpm, 15.7 cpm and 8.2 cpm per additional hour of evening daylight; these changes translate into standardised effect sizes of 0.07, 0.06 and 0.03, respectively. The equivalent effect sizes in terms of percent of daily time spent in MVPA were 0.19%, 0.20% and 0.05% per additional hour of evening daylight, corresponding to around 1.6 minutes, 1.7 minutes and 0.4 minutes respectively. By contrast, there was little or no consistent evidence of associations with evening daylight in the American samples or in the Madeiran and Brazilian samples, with standardised effect sizes ranging from -0.02 to +0.01 and in all cases non-significant. A post-hoc univariable meta-regression analysis provided some evidence that the smaller magnitude of the associations in these latter settings might reflect their higher maximum temperatures (adjusted R^2^ = 51%, p = 0.01: see Additional file 1: Figure A2 and accompanying text).

Although associations with evening daylight varied markedly between settings, there was less convincing evidence for interactions with the child’s characteristics (Figure 4, plus Additional file 1: Figures A3-A4). This lack of an interaction was clearest for weight status and maternal education, with neither variable showing any significant interaction in any of the five study settings, and with the overall pooled effect sizes also non-significant. By contrast, the non-significant pooled interaction terms for sex and age were harder to interpret as in both cases there was some evidence of between-study heterogeneity (0.01 ≤ p ≤ 0.02). With respect to sex, this heterogeneity reflected the fact that the association between evening daylight and physical activity tended to be stronger in boys than in girls in some European and English samples, but this was not the case in the other settings (Additional file 1: Figure A3). With respect to age, there was no very obvious pattern: the magnitude of the association with evening daylight was greater among younger children in Denmark, was greater among older children in Australia, and did not differ according to age in the remaining three settings for which sufficient data were available.

## Discussion

Among 23,000 school-age children from 9 countries, we found strong evidence that longer evening daylight was associated with a small increase in daily physical activity, even after adjusting for weather conditions. Consistent with a causal interpretation, the magnitude of this associations was largest in the late afternoon and early evening, including when the same child was measured immediately before and after the clocks went forward or back. These associations were, however, only consistently observed in the European and Australian samples. There was inconsistent evidence that the magnitude of the association with evening daylight was greater in boys; no evidence of any differences in the magnitude of the association according to weight status or maternal education; and inconsistent findings for interactions with age.

### Limitations and directions for future research

This study substantially extends previous analyses of some subsets of this data, which have at most only provided a relatively brief examination of physical activity differences by season [29, 30]. It also addresses several recognised limitations of the existing literature [14], including small sample sizes, inconsistent accelerometer protocols and little or no examination of interactions with factors such as age, sex or weight status. In addition, our large sample size allowed us to use the bi-annual changing of the clocks as a natural experiment, and to show significant differences in children’s activity levels either side of the clock change. This observation considerably strengthens the case for a causal interpretation of the association between evening daylight and physical activity, as does the fact that the fastest decrease in children’s evening physical activity coincided with sunset throughout the year.

This study does, however, also have several important limitations. First, our data were largely cross-sectional rather than longitudinal: although we could follow the same child across the week when the clocks changed, we could not follow children across a full year. We have, however, no reason to believe that children sampled at different times of the year differed systematically within or between studies.

A second set of limitations involves data not available to us. For one thing, although we adjusted for observed weather conditions on each day of measurement, the timing of some physically active events may instead reflect *expected* weather conditions (e.g. some schools may routinely schedule sports days on summer afternoons in the hope that it will be warm and dry). Failing to adjust for such social expectations may mean that our effect estimates are still subject to some residual confounding by weather, and this may partly account for why small differences in activity levels were seen as early as 14:00 on weekend/holidays. In addition, we lacked any data on the behavioural mediators of the observed activity differences. As such, we cannot examine how far one can generalise the findings of one previous, small English study which found that associations between day length and activity levels were largely mediated by outdoor play [16]. This is one useful direction for future research, perhaps particularly as it becomes increasingly possible to substitute or complement detailed activity diaries with data from global positioning systems (GPS) monitors [31]. We also lacked systematic information on area-level factors such as neighbourhood safety or the availability of green space which might plausibly moderate the effect of evening daylight upon physical activity; again, this would be one useful direction for future research. Also of interest would be an examination of how a wider range of behaviours vary with daylength; these were largely beyond the scope of what is possible in the ICAD database, although the lack of any association between time of sunset and accelerometer weartime provides some indirect evidence against an effect of evening daylight on children’s duration of sleep.

Finally, most of our study populations came from Europe and almost all came from high-income settings, meaning that more research would be needed to establish how far the observed associations apply across other settings. Our data do, however, give some hints that daylight saving measures might not increase activity in hot settings, perhaps because high temperatures may inhibit summertime activity.

### Implications for policy and practice

The British parliament recently debated a Bill proposing new daylight saving measures which would shift the clocks forward by one additional hour year round [10]. If the adjusted, pooled effect size we observed in this study were fully causal, one would expect the proposed daylight savings measures to generate a 0.06 standard deviation increase in the total physical activity of English children, corresponding to an estimated 1.7 extra minutes of MVPA per day. The equivalent standardised effect sizes in mainland Europe and in Australia were 0.07 and 0.03, respectively. As such, introducing additional daylight saving measures in any of these settings would be likely only to have a small-to-very-small average effect upon each child. Such measures would, however, have far greater reach than most other potential policy initiatives, with these small average effects applying every day to each and every child in the country. This is important because even small changes to the population mean can have important public health consequences [8]. Moreover, although these population-level effect sizes are small in absolute terms, the English and mainland European effect estimates actually compare relatively favourably to individual-level approaches, despite the latter generally being much more intensive (and expensive). For example, one recent meta-analysis of 22 randomised controlled comparisons reported a standardised pooled effect size of 0.12 (95% CI 0.04, 0.20) for interventions seeking to promote child or adolescent physical activity [32].

Notably, the association between longer evening daylight and higher physical activity was observed irrespective of weight status or maternal education. This contrasts with one previous Australian survey in which daylight savings measures seemed to have the largest effects among normal weight adults from socio-economically advantaged groups [33]. Further research in adults would be useful to confirm this finding, ideally using objectively-measured activity data. Speculatively, however, a relatively wide range of children may respond to longer evening daylight by playing more outdoors whereas among adults the effect may primarily be confined to the groups with the highest propensity to exercise.

## Conclusions

This study provides the strongest evidence to date that, in European and Australian settings, evening daylight plays a causal role in increasing physical activity in the late afternoon and early evening – a period which has been described as the ‘critical hours’ for children’s physical activity [34]. In these settings, it seems possible that additional daylight saving measures could shift mean population child physical activity levels by an amount which, although small in absolute terms, would not be trivial relative to what can feasibly be achieved through other approaches. Moreover, our findings also suggest that this effect might operate in a relatively equitable way. As such, while daylight savings proposals such as those recently considered in Britain would not solve the problem of inadequate levels of child physical activity, this paper indicates that they could represent a small step in the right direction.

## Electronic supplementary material

Additional file 1: **Additional methods and results; presentation of fuller details on the studies included in the analyses, and additional results.** (PDF 759 KB)

## Abbreviations

CI: Confidence intervals
cpm: Counts per minute
ICAD: International Children’s Accelerometry Database
MVPA: Moderate-to-vigorous physical activity.
