# Supplementary material for: Daylight saving time as a potential public health intervention: an observational study of evening daylight and objectively-measured physical activity among 23,000 children from 9 countries
Source: Int J Behav Nutr Phys Act. 2014 Oct 23;11:84. doi: 10.1186/1479-5868-11-84 (PMC4364628; doi:10.1186/1479-5868-11-84)
Supplement: Supplementary file 1 — Additional file 1: Additional methods and results; presentation of fuller details on the studies included in the analyses, and additional results. (PDF 759 KB) [file 12966_2014_940_MOESM1_ESM.pdf]

Additional information on methods

Table A1: ICAD studies: country of origin, design and characteristics of study participants included in the present analyses

No.	Full name	Short name	Country	Design (waves included)	Years	Mother education available (% with data)	N	% female	Age
1	Denmark European Youth Heart Study	Denmark EYHS	Denmark	Longitudinal (2 waves)	1997 - 2004	Yes (94%)	1364	56%	8 - 16
2	Copenhagen School Child Intervention Study	Denmark CSCIS	Denmark	Natural experimental	2001 - 2003	No	667	48%	5 - 11
3	Estonia European Youth Heart Study	Estonia EYHS	Estonia	Cross-sectional	1998 - 1999	Yes (90%)	656	55%	8 - 16
4	Kinder-Sportstudie Study	Switzerland KISS	Switzerland	RCT (baseline only)	2005	Yes (95%)	404	52%	6 - 12
5	Norway European Youth Heart Study	Norway EYHS	Norway	Cross-sectional	1999 - 2000	Yes (93%)	384	50%	8 - 10
6	Avon Longitudinal Study of Parents and Children	ALSPAC	England	Longitudinal (2 waves)	2003 - 2007	Yes (82%)	6593	53%	10 - 15
7	Personal and Environmental Associations with Children's Health	PEACH	England	Longitudinal (2 waves)	2006 - 2009	Yes (45%)	1241	54%	9 - 12
8	Sport, Physical activity and Eating behaviour: Environmental Determinants in Young people	SPEEDY	England	Cross-sectional	2007	No	1967	56%	9 - 11
9	Children's Health and Activity Monitoring for Schools, UK	CHAMPS UK	England	Cross-sectional	2006 - 2007	No	483	50%	5 - 16
10	Children Living in Active Neighbourhoods	CLAN	Australia	Longitudinal (3 waves)	2001 - 2006	Yes (96%)	1117	54%	5 - 16
11	Healthy Eating and Play Study	HEAPS	Australia	Longitudinal (2 waves)	2002 - 2006	Yes (97%)	1342	53%	5 - 15
12	Iowa Bone Development Study	Iowa Bone Study	USA	Longitudinal (4 waves)	1998 - 2007	Yes (84%)	579	51%	5 - 14
13	Project Trial of Activity for Adolescent Girls	Project TAAG	USA	RCT (baseline + 2 follow-up waves for controls)	2002 - 2006	Yes (74%)	4724	100%	10 - 16
14	Portugal (Madeira) European Youth Heart Study	Madeira EYHS	Portugal (Madeira)	Longitudinal (2 waves)	1999 - 2008	Yes (52%)	1214	50%	8 - 17
15	Pelotas 1993 Birth Cohort	Pelotas, Brazil	Brazil	Cross-sectional	2006 - 2007	No	453	47%	12 - 14

See Sherar *et al.*(2011)for further details about the 15 studies. RCT=randomised controlled trial; only baseline samples used in RCTs except in Project TAAG where follow-up repeat-cross-sectional samples from control schools also included, because in this study it was possible to distinguish intervention and control children.

Reference: Sherar LB, Griew P, Esliger DW*, et al.* International children's accelerometry database (ICAD): design and methods. *BMC Public Health* 2011;**11**:485.

Table A2: Raw education categories underlying re-coded maternal education data

	Study	Raw maternal education categories	Re-coded categories
1	Denmark EYHS	• Primary<br>• Secondary<br>• EFG basic<br>• EFG 2	Up to high school
		• Apprentice<br>• Short further education	College / vocational
		• Medium or long-term further education	University level
3	Estonia EYHS	• < 8 years (basic education)<br>• 9-11 years (secondary education)	Up to high school
		• 11-13 years (specialised secondary)	College / vocational
		• Higher not graduated<br>• Higher education	University level
4	Switzerland KISS	• Mandatory schooling only<br>• Apprenticeship/informal education of under 2 years	Up to high school
		• Formal apprenticeship of 2-4 years	College / vocational
		• University or equivalent	University level
5	Norway EYHS	• Primary<br>• Secondary	Up to high school
		• University / Norwegian folk school	University level
6	ALSPAC	• No qualifications<br>• O-levels, CSEs or GCSEs<br>• A-levels<br>• Qualifications in shorthand, typing or other skills	Up to high school
		• Apprenticeship<br>• State enrolled nurse or state registered nurse<br>• City & Guilds intermediate or final technical qualification	College / vocational
		• Teaching qualification<br>• University degree	University level
7	PEACH	• GCSE<br>• A-level	Up to high school
		• First degree<br>• Higher degree	University level
10 & 11	CLAN and HEAPS	• Never attended school<br>• Primary school<br>• Some high school or completed high school	Up to high school
		• Technical or trade school certificate	College / vocational
		• University or tertiary qualification	University level
12	Iowa Bone Study	• Did not finished high school<br>• High school diploma	Up to high school
		• Some college<br>• 2 years college degree	College / vocational
		• 6-4 years college degree<br>• Graduate or professional school	University level
13	Project TAAG	• Did not finish high school<br>• Finished high school	Up to high school
		• Vocational training<br>• Some college	College / vocational
		• Graduated college or university<br>• Professional training beyond 4 years	University level
14	Madeira EYHS	• No school<br>• 1st grade through to 10th grade	Up to high school
		• 11 or 12th grade	College / vocational

Additional analyses

Figure A1: Adjusted average physical activity across the hours of the day, according to the time of sunset: including confidence intervals

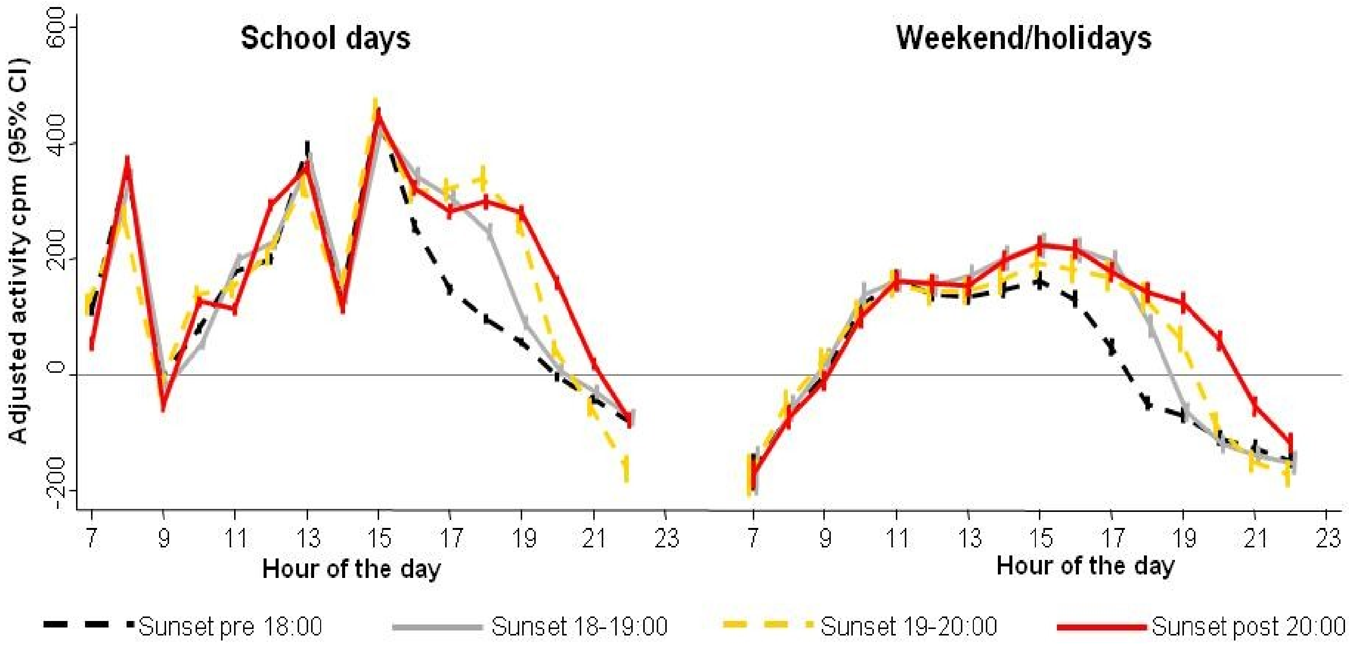

CI=confidence interval, cpm=counts per minute.

Figure A2: Association between maximum temperature during the measurement period of each study and the magnitude of the effect of evening daylight upon physical activity (N=15 studies)

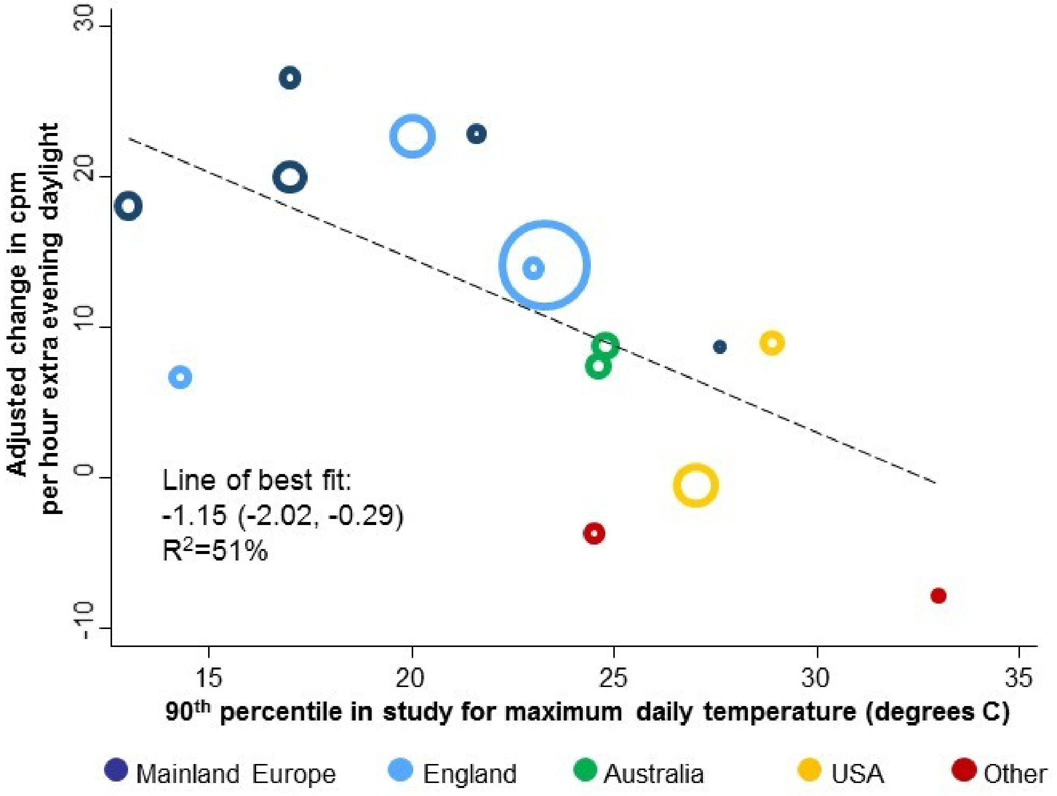

Larger circles represent more precise estimates from larger studies, with the size of the circles equal to the inverse of the within-study variance. The dashed line indicates the line of best fit from univariable random effects meta-regression.

In the above figure, we used meta-regression (Harbord & Higgins, 2008) to test the hypothesis that the magnitude of the association with evening daylight would be smaller in very hot settings, operationalising temperature as the 90th centile for daily maximum temperature during the observation period. This meta-regression analysis was post-hoc insofar as it was not pre-planned; it was, however, the only post-hoc meta-regression analysis we conducted.

Reference: Harbord H, Higgins JP. Meta-regression in Stata. *The Stata Journal* 2008;**8**:493–519.

Figure A3: Interaction between (a) sex and (b) age and hours of evening daylight, by study population†

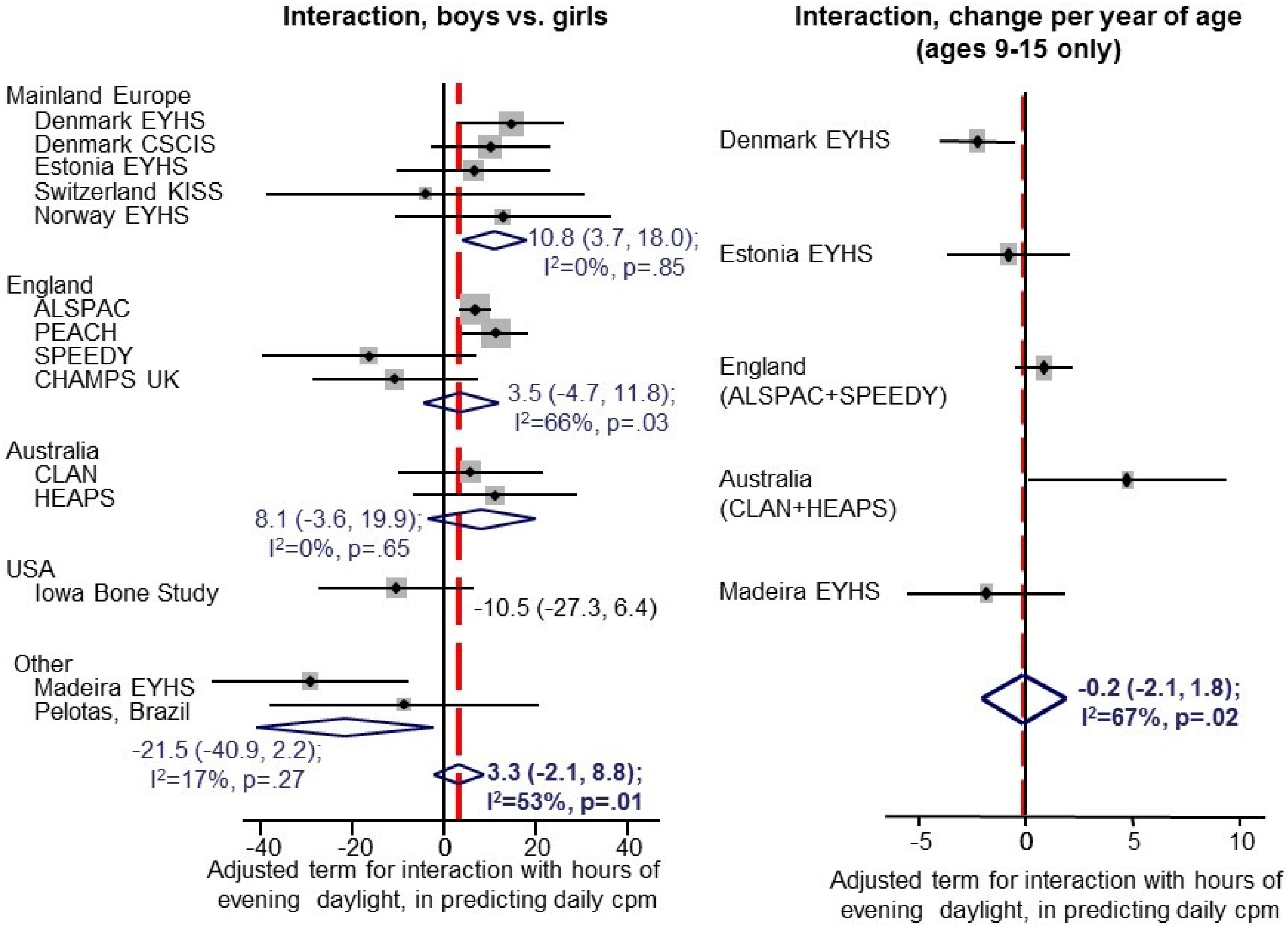

† For analyses by age, study populations from the same country were grouped in England and Australia to generate a sufficient sample size spanning the range 9-15; countries without a sample spanning this range are not presented. Random-effects pooled estimates are presented by country/region, with the red dotted line indicating the position of the overall pooled effect size. Effect sizes and 95% confidence intervals are shown following tests for interaction, with the adjusted interaction term representing the difference that the interaction variable (e.g. sex) makes to the effect size for evening daylight upon total daily activity measured in cpm.

Figure A4: Interaction between (a) weight status and (b) maternal education and hours of evening daylight, by study population

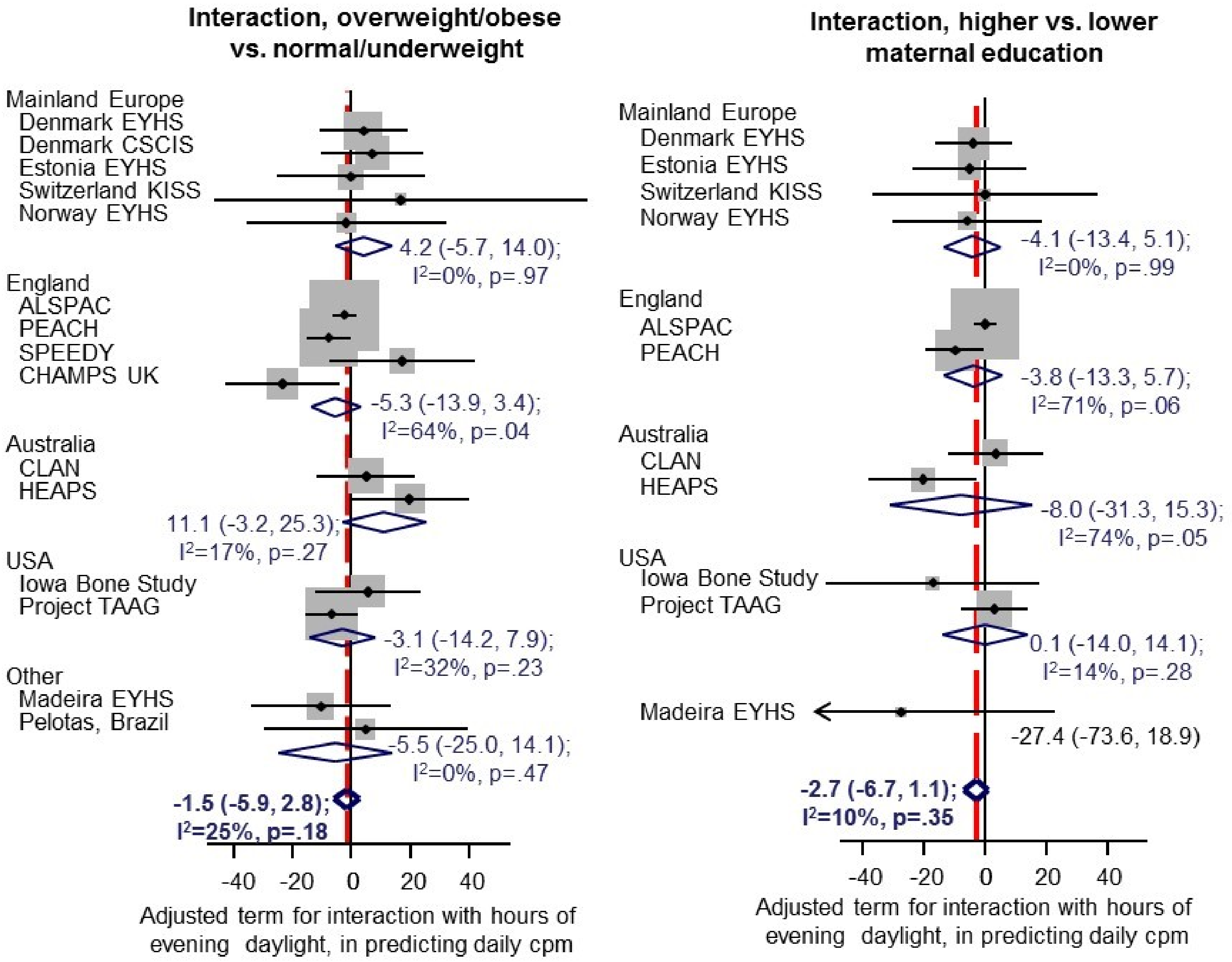

Random-effects pooled estimates presented by country/region, with the red dotted line indicating the position of the overall pooled effect size. Effect sizes and 95% confidence intervals are shown following tests for interaction, with the adjusted interaction term representing the difference that the interaction variable (e.g. overweight/obese weight status) makes to the effect size for evening daylight upon total daily activity measured in cpm.
